# Test-retest reliability of stride time variability while dual tasking in healthy and demented adults with frontotemporal degeneration

**DOI:** 10.1186/1743-0003-8-37

**Published:** 2011-07-11

**Authors:** Olivier Beauchet, Ellen Freiberger, Cedric Annweiler, Reto W Kressig, Francois R Herrmann, Gilles Allali

**Affiliations:** 1 Department of Internal Medicine and Geriatrics, Angers University Hospital (4 rue larrey), Angers (F-49933), France; 2 Institut fur Sportwissenschaft und Sport, Friedrich-Alexander-Universitaet Erlangen-Nuernberg, (Gebbertstr. 123b), Erlangen (91058), Germany; 3 Department of Acute Geriatrics, Basel University Hospital and University of Basel, (Spitalstrasse 21/Petersgraben 4), Basel (4031), Switzerland; 4 Department of Rehabilitation and Geriatrics, Geneva University Hospitals of Geneva, (12 chemin du pont bochet), Geneva (1226), Switzerland; 5 Department of Neurology, Geneva University Hospital of Geneva, (rue Micheli-du-Crest 24), Geneva (1205), Switzerland

## Abstract

**Background:**

Although test-retest reliability of mean values of spatio-temporal gait parameters has been assessed for reliability while walking alone (i.e., single tasking), little is known about the test-retest reliability of stride time variability (STV) while performing an attention demanding-task (i.e., dual tasking). The objective of this study was to examine immediate test-retest reliability of STV while single and dual tasking in cognitively healthy older individuals (CHI) and in demented patients with frontotemporal degeneration (FTD).

**Methods:**

Based on a cross-sectional design, 69 community-dwelling CHI (mean age 75.5 ± 4.3; 43.5% women) and 14 demented patients with FTD (mean age 65.7 ± 9.8 years; 6.7% women) walked alone (without performing an additional task; i.e., single tasking) and while counting backward (CB) aloud starting from 50 (i.e., dual tasking). Each subject completed two trials for all the testing conditions. The mean value and the coefficient of variation (CoV) of stride time while walking alone and while CB at self-selected walking speed were measured using GAITRite^® ^and SMTEC^® ^footswitch systems.

**Results:**

ICC of mean value in CHI under both walking conditions were higher than ICC of demented patients with FTD and indicated perfect reliability (ICC > 0.80). Reliability of mean value was better while single tasking than dual tasking in CHI (ICC = 0.96 under single-task and ICC = 0.86 under dual-task), whereas it was the opposite in demented patients (ICC = 0.65 under single-task and ICC = 0.81 under dual-task). ICC of CoV was slight to poor whatever the group of participants and the walking condition (ICC < 0.20), except while dual tasking in demented patients where it was fair (ICC = 0.34).

**Conclusions:**

The immediate test-retest reliability of the mean value of stride time in single and dual tasking was good in older CHI as well as in demented patients with FTD. In contrast, the variability of stride time was low in both groups of participants.

## Background

Stride time variability (STV), as measured by the coefficient of variation (CoV), is considered as a marker for the control of limb-coordinated movements [1,2]. In terms of gait control, a low variability of STV while steady state walking reflects an automatic process requiring minimal attention, whereas a high variability is related to major attention involvement [3]. Dual-task paradigms are used by clinicians to evaluate the cortical involvement in gait control [4]. Changes in gait performance while performing an attention-demanding task compared to walking alone result from interference caused by competing demands for attention resources and depend in part on the efficiency of executive functions [4-6].

Demented older adults demonstrate greater gait impairments than those expected with normal aging process [7,8]. In particular, frontal lobe dysfunction has been associated with gait disorders in dementia [5,6,9]. Thus, exploring gait variability under single and dual tasking in demented patients with frontotemporal degeneration (FTD) and in cognitively healthy individuals (CHI) might improve our understanding of higher-level gait disorders in dementia.

Counting backward (CB) is an attention-demanding task frequently used in dual task paradigm involving gait [4]. Compared with other attention-demanding tasks used in dual-task paradigms involving gait, CB has been previously associated with high gait changes in demented patients with impaired executive functions [5,6,9]. For instance, it has been reported that patients with Alzheimer's disease (AD) or mixed dementia presenting with impaired executive functions exhibited an increase in STV during single and dual tasking compared to non-demented counterparts [5,9]. It has also been reported in a group of demented patients with impaired executive functions that changes in CoV of stride time while CB reflected the best dual-task interference [6].

STV may be interpreted as surrogate marker for gait control only after it has been determined if dual-task-related changes of STV are due to a pathological process affecting older adults, to a normal biological variability following consecutive gait measures from trial to trial or to a measurement error. Although test-retest reliability of mean values of spatio-temporal gait parameters has been assessed for reliability while walking alone, little is known on the test-retest reliability of gait variability while dual tasking [10-12]. Recently, Hollman et al. showed in healthy older adults that immediate test-retest reliability for variability in stride velocity was poor while dual tasking [11]. There is a lack of data for the test-retest reliability of STV under single and dual-task conditions. The objective of this study was to examine immediate test-retest reliability of STV while single and dual tasking in CHI and in demented patients with FTD.

## Methods

Out of 80 community-dwelling CHI participating in a large fall prevention program in Erlangen, Germany, 69 (90.0%) CHI (mean age 75.5 ± 4.3; 43.5% women) with at least 3 consecutive measured gait cycles were included in this study, with respect to the European guidelines for clinical applications of spatio-temporal gait analysis in older adults [13]. In addition, 14 consecutive demented patients (mean age 65.7 ± 9.8 years; 6.7% women) with FTD followed in a memory center in Paris, France, were also included in the study. The selection of CHI has been described elsewhere in details [14]. In summary, CHI were drawn from a health insurance company membership database. They were excluded if they were unable to walk independently, were under 70 years of age or had cognitive impairment. For the demented patients, diagnosis of FTD was based on the revised Lund and Manchester criteria [15]. Dementia severity was measured with the Mini-Mental State Examination (MMSE) [16] and impairment in EF using the Frontal Assessment Battery (FAB) [17]. A FAB score of 18 indicates normal executive functions. The mean duration of disease for FTD group was 4.2 ± 1.9 years. Demented patients took 3.9 ± 2.5 drugs per day on average. The mean MMSE score (/30) and the mean FAB score (/18) were respectiveley 23.3 ± 6.6 and 12.6 ± 3.8. Exclusion criteria for FTD consisted in extrapyramidal rigidity of the upper limbs with a score above 2, based on item 22 of the Unified Parkinson's Disease Rating Scale motor score (UPDRS)-motor score [18]; acute medical illness in the past 3 months; neurological and psychiatric diseases except dementia; severe orthopaedic or rheumatologic conditions affecting normal walking, as well as use of walking aids. Written informed consent was either obtained from the subject or from their legal representative in case of cognitive decline. The study was conducted in accordance with the ethical standards set forth in the Helsinki Declaration (1983). Each local ethics committee approved the project.

The mean value and the CoV (CoV = [standard deviation/mean] × 100) of stride time while walking alone (i.e., single tasking) and while counting backward (CB) aloud starting from 50 (i.e., dual tasking) were collected. Gait measurements were made according to the guidelines for clinical applications of spatio-temporal gait analysis in older adults [13]. The mean value and the CoV of stride time were measured at self-selected walking speed and while steady state walking using GAITRite^® ^and SMTEC^® ^footswitches systems which are two validated devices providing similar measures of stride time [19]. The GAITRite^® ^system is an electronic walkway-integrated, pressure-sensitive electronic surface connected to a personal portable computer via an interface cable. The SMTEC^® ^footswitches system is a pair of innersoles fitted inside the subject's shoes. Each innersole contains 2 independent footswitches placed at the heel and the toe, which are linked to a portable data logger worn at the waist. To familiarize participants using the SMTEC^® ^system, each participant performed one practice walk before recording.

The GAITRite^® ^and the SMTEC^® ^footswitches systems used the same definition of stride time which was the time elapsed between the first contacts of two consecutive footfalls of the same foot expressed in ms. Walking trials were recorded on a 3.5-meter walkway for GAITRite^® ^and 10-meters walkway for SMTEC^®^. To assure measuring of steady-state walking among CHI, participants started walking 2 meters before the active measuring electronic surface area and stop 2 meters after. In the group of demented patients with FTD, stride time parameters were collected on a 14-meter long walkway but were analyzed only over a distance of 10 meters. The first and last 2 meters corresponding to the acceleration and deceleration phase of each pass were excluded from analysis. All participants were asked to perform the walking tasks without prioritizing walking or cognitive task. Before testing, a trained evaluator gave standardized verbal instructions regarding the test procedure with a visual demonstration of the walking test. Each subject completed two trials for all the testing conditions. The walking trial was performed in a well-lit environment. The participants walked at their self-selected speed and wore their own footwear.

Immediate test-retest reliability of STV was evaluated comparing the first and the second trial performed while walking alone and while CB using intraclass correlation coefficient (ICC). Separated analyses were used for single and dual task condition. Using Landis and Koch interpretation of agreement, an ICC > 0.80 indicated almost perfect reliability, 0.61-0.80 substantial, 0.41-0.60 moderate, 0.21-0.40 fair, 0.00-0.20 slight, below 0.0 poor [20]. P-values less than 0.05 were considered as statistically significant. All statistics were calculated using the Stata Statistical Software, version 11.1.

## Results

All participants were able to complete single and dual-task walking, without falling. As shown in Table 1, ICC of mean value in CHI while single and dual tasking were higher than ICC of demented patients with FTD and indicated perfect reliability (ICC > 0.80). Reliability of mean value was better while single tasking than dual tasking in CHI (ICC = 0.96 under single-task and ICC = 0.86 under dual-task), whereas it was the opposite in demented patients with FTD (ICC = 0.65 under single-task and ICC = 0.81 under dual-task). ICC of CoV was slight to poor, whatever the group of participants and the walking condition (ICC < 0.20), except while dual tasking in demented patients where ICC was fair (ICC = 0.34).

**Table 1:** Mean value and standard deviation of stride time parameters for two consecutively repeated trials while walking alone awhile walking with counting backward among cognitively healthy individuals (n = 69) and demented patients with frontotemporal dementia (n = 14)

	Stride time					
	
	Mean value		ICC	Coefficient of variation		ICC
				
	Trial 1	Trial 2		Trial 1	Trial 2	
Cognitively healthy individuals						
Walking alone	1065.6 ± 105.3	1061.82 ± 104.0	0.96	1.3 ± 1.0	1.1 ± 0.8	-0.01
Walking with counting backward	1086.9 ± 183.5	1090.5 ± 225.1	0.86	1.7 ± 1.4	2.0 ± 3.3	0.11
Demented participants						
Walking alone	1108.6 ± 90.2	1102.9 ± 83.4	0.65	5.0 ± 2.5	6.7 ± 4.6	-0.12
Walking with counting backward	1263.6 ± 124.5	1302.9 ± 131.2	0.81	7.6 ± 6.7	10.5 ± 9.3	0.34

±: Standard deviation.

ICC: Intra Class Coefficient Correlation

## Discussion

Our results showed that the immediate test-retest reliability of the mean value of stride time was perfect (i.e., ICC > 0.80), higher than the reliability of the CoV and better in CHI than in demented patients with FTD. In contrast, the reliability of the CoV was slight to poor in both groups of participants, except while dual tasking in the group of demented patients with FTD by whom it was fair. In addition, the reliability of both stride parameters was better while dual tasking compared to single tasking in demented patients with FTD but not in CHI.

The very good immediate test-retest reliability of mean value of stride time while single tasking showed in the studied sample of CHI and demented patients with FTD is consistent with the literature. Indeed, previous studies showed a high ICC for most temporo-spatial gait parameters including stride time while walking alone at usual walking speed [21-23]. Immediate test-retest reliability of mean value of spatio-temporal gait parameters while dual tasking has been few studied compared to the single task condition. Our results highlight a perfect reliability of the mean value (i.e., ICC > 80) while dual tasking based on Landis and Koch interpretation of agreement [20] but under the reliability of walking alone, except for demented patients. Like our results, it has been recently shown that the ICC of the mean value of velocity was slightly lower than the ICC of mean value while single tasking in CHI [11]. In addition, we showed that ICC of the mean value of stride time was better in CHI than in demented patients with FTD. This result is also in concordance with the fact that walking patterns of people with dementia are more variable than those seen in normal ageing. This mainly illustrates that, among demented patients, increased variability occurs in both spatial and temporal gait measures leading to lower immediate test-retest reliability than found among CHI [7,8,12].

In contrast to the mean value, little is known about the test-retest reliability of stride-to-stride variability. Our results show that the immediate reliability of the CoV of stride time while single and dual tasking is poor, which is in concordance with the few previous published data. For instance, Hollman et al. recently reported among healthy older adults that immediate test-retest reliability for variability in stride velocity was poor while dual tasking [11]. This low reliability of CoV of stride time indicates that the measurement lacks of consistency for immediate test retest. Poor measurement reliability generally comes from three main sources which are the innate random variability from trial to trial, the gait speed, and/or a measurement error. Because of the very good immediate test-retest reliability of the mean value of stride time, the methodology we used (i.e., computerized walkway) for measuring gait characteristics and a measurement error may be excluded. Variation of gait speed between trials could explain the low reliability of CoV of stride time. Indeed, it has been shown that one of the main factors influencing the STV is walking speed [24-26]. An increase in gait speed has been associated with an increase in stride time variability. The fact that we found a good reliability of mean value of stride time ranging from perfect to substantial suggests that participants, whatever their cognitive status, did not vary their walking speed dramatically from one trial to another. Therefore, the modest reliability of gait variability measures cannot be attributed to true between-trial changes in walking speed, but seem to be related to innate random variability.

Whilst the reliability of CoV was slight to poor in most case, our results highlight that the reliability while dual tasking in demented patients with FTD was higher compared to single tasking (ICC = 0.34 while walking with CB versus ICC = -0.12 while walking alone) and compared to CHI (ICC = 0.11). One explanation could be a practice effect related to the repetition of trials. We showed that demented patients had a higher mean value of CoV while dual tasking compared to CHI and compared to walking alone, which is in concordance with previously published data. We have earlier shown that CB in demented participants with impaired EF provoked severe perturbation in gait control resulting in an increased CoV of stride time compared to the mean value while dual tasking [5,6,9]. Thus, the fact that demented patients with FTD had a higher mean value of CoV than in CHI suggest that they were disturbed by CB as well during the first as the second gait trials with no motor skill effect. We observed a similar effect with the mean value of stride time.

The main limitation of our study was the short length of the walkway used to assess STV. The European GAITRite network group recommends the highest number of gait cycles possible from a practical standpoint, with a minimum of three consecutive gait cycles [13]. Another limitation could be in relation with the articulo-motor components of enumerated figures which is different in German and in French. However, it has been shown that dual task-related stride time changes while CB in demented participants with frontal lobe dysfunction could not be explained by the articulo-motor components of speech [27].

## Conclusions

Measurements of stride time variability had low immediate test-retest reliability in older CHI as well as in demented patients with FTD either in single or dual task condition. In contrast, the reliability of the mean value was good in both groups. This result suggests a normal biological variability for stride time variability between two immediate consecutive gait measurements.

## Competing interests

All authors have no conflicts of interest. There were not any financial and personal relationships with other people or organization that could influence this study.

### Financial Disclosure(s)

No authors have relevant financial interest in this manuscript.

### Authors' contributions

OB has full access to the data in the study and takes responsibility for the integrity of the data and the accuracy of the data analyses; study concept and design: OB and GA; acquisition of data: GA and EF; analysis and interpretation of data: OB, GA, CA and FH; drafting of the manuscript: OB, GA, CA and EF; critical revision of the manuscript for important intellectual content: FH and RK; obtained funding: not applicable; statistical expertise: FH; administrative, technical, or material support: OB; study supervision: OB and GA.

All the authors (OB, EF, CA, RWK, FRH, GA) have participated in the research reported, have seen and approved the final version of the manuscript, and have agreed to be an author of the paper.

## Funding

Gilles Allali was supported by a grant from the Swiss National Science Foundation (No 33CM30-124115).
